# Exploring the role of nutritional strategies to influence physiological and cognitive mechanisms in cold weather operations in military personnel

**DOI:** 10.3389/fphys.2025.1539615

**Published:** 2025-02-28

**Authors:** Dagmar J. Smid, Lisa Klous, Sam B. Ballak, Milène Catoire, Iris M. De Hoogh, Femke P. M. Hoevenaars

**Affiliations:** ^1^ Research group Microbiology and Systems Biology, Netherlands Organization for Applied Scientific Research (TNO), Leiden, Netherlands; ^2^ Research group Human Performance, Netherlands Organization for Applied Scientific Research (TNO), Soesterberg, Netherlands

**Keywords:** cold mitigation, nutrition, micronutrients, cold weather injuries, military performance

## Abstract

**Introduction:**

As a basis for performance optimal nutritional balance is key to keep the body functioning at homeostatic capacity. When environmental circumstances become challenging such as in a cold environment extraordinary performance is requested specifically for physiological (i.e., vascular response, diet induced thermogenesis, immune response), and cognitive mechanisms (i.e., cognitive function, psychological and cognitive wellbeing) of the human body. In this review we describe which nutritional strategies could enhance military performance in the cold by mitigation of CWIs.

**Methods:**

We will first describe how exposure to cold affects the physiological or cognitive mechanisms itself and then we will explain how nutrition can be used to optimize these affected mechanisms. We will discuss long-term nutritional solutions preventing shortfalls and potential direct quick fixes for physiological and cognitive mechanisms.

**Results:**

For optimal functioning of the immune system and infection prevention, absence of micronutrient deficiencies is key and should be pursued amongst military personnel. For the effectivity of PUFA’s, Echinacea purpurea and probiotics in immune functioning, more research is needed in the CWO context. A multitude of micronutrients (i.e., nitrate, L-citrulline, L-arginine) appears to be able to enhance vasodilation, perhaps partially offsetting the detrimental effect of cold on peripheral blood circulation. Although the direct effect of diet induced thermogenesis is small in comparison to being physically active, it is of interest to investigate the effects of adding a combination of spices to the rations, such as capsaicin from red pepper, cinnamon, ginger, and menthol. Also, of interest for stimulation of thermogenesis are caffeine, and polyphenolic compounds. Caffeine and tyrosine supplementation 1 h, resp. 2 h before a cognitively demanding task during CWOs could be used to mitigate decreases in cognitive performance. Alternatives that are of interest, but need more research, include chocolate polyphenols and omega-3 fatty acids.

**Conclusion:**

Even though some recommendations can be provided, it is evident that much information regarding the effectiveness and application of micronutrients in cold weather operations is still lacking. More focus should be placed on investigating (micro)nutritional solutions, practical feasibility, and implementation in operational military personnel to better understand the magnitude of the possible benefits in cold conditions.

## 1 Introduction

Military personnel work in demanding environments and are exposed to challenging physical and psychological stressors. Many factors can optimize deployability when exposed to demanding environmental conditions, such as cold. Specific education and training on appropriate behavior, amongst others, is undoubtedly of utmost importance (Sullivan-Kwantes et al., 2021; Castellani et al., 2006; Klous et al., 2024). Nutrition plays an important part in minimizing reductions in deployability under these conditions (Margolis and Pasiakos, 2023). Yet, dietary intake is typically hampered in military cold weather operations (CWOs) (Margolis et al., 2014; Margolis et al., 2016), meaning that nutritional guidelines are not met by service personnel. At the same time, during CWOs, the body’s energy demand is increased, mainly due to the type of activities that are usually performed in the cold (i.e., walking in snow and skiing) (Castellani et al., 2017). Energy requirements have been reported to go as high as 5,000–7,000 kcal/day (Margolis and Pasiakos, 2023). Potential nutritional deficiencies could reduce the physical and cognitive capacity of service personnel in these environments, reducing deployability in CWOs (Margolis and Pasiakos, 2023). Recent geopolitical developments require optimization of CWOs education and training and a search for innovative solutions to maintain deployability in these environments.

One way to mitigate performance deterioration in CWOs is to prevent caloric deficits by increasing caloric intake (Margolis and Pasiakos, 2023). Caloric deficits could negatively affect thermoregulation, jeopardizing immune system function as well as physical and cognitive performance (Pasiakos, 2020). This necessitates an increase in caloric intake, preferably using energy-dense foods to improve palatability (Margolis and Pasiakos, 2023). However, nutritional interventions (e.g., adding snacks) aimed at increasing caloric intake turned out to be unsuccessful (Margolis et al., 2014; Margolis et al., 2016; McClung et al., 2013). Anecdotally, in military practice, it is often challenging to increase caloric intake due to not being hungry, limited time to eat, and difficulties in preparing food. Considering these challenges, alternative nutritional strategies in cold environments may be relevant to explore. In addition, as cold per se negatively influences several bodily mechanisms, it is of relevance to focus on the potential of nutritional strategies to promote optimal physiological functioning and decrease cold weather injuries (CWIs). Mechanisms affected by cold include physiological mechanisms such as the vascular response, dietary-induced thermogenesis, and immune response, as well as cognitive mechanisms. These are all essential mechanisms for effective military deployment in CWOs and improvements in these mechanisms may decrease risk of CWIs. Therefore, understanding the mechanism by which nutritional strategies could potentially enhance performance and operational deployability–partly by counteracting the detrimental influences of cold – in CWOs is crucial.

Therefore, this review will focus on the effects of nutritional strategies on several physiological and cognitive mechanisms to enhance military performance in CWOs. First, the effect of cold exposure on the physiological or cognitive mechanism itself will be discussed, followed by an explanation of the effects of nutrients and bioactive compounds on these mechanisms. In discussing potential nutritional strategies, a distinction is made between strategies with an acute effect during CWOs and maintaining nutritional status for optimal physiological processes (for long term benefits). The review concludes with recommendations for military performance enhancement using nutritional strategies in CWOs.

## 2 Physiological mechanisms

### 2.1 Immune function

The immune system can be affected by multiple stressors, including cold exposure. Cold exposure leads to a greater incidence of upper respiratory tract infection (URTI) in military personnel (Mäkinen et al., 2009). Furthermore, cold exposure – with or without exercise – may cause leukocytosis (i.e., high white blood cell count), showing increases in circulating neutrophils, lymphocytes and NK cells (Walsh and Whitham, 2006). In a study executed in the Chilean Army, immunological changes due to hypothermic stress were identified, showing increases in immune cells, including leukocytes, neutrophils, basophils, monocytes, and platelets (Nieto Jimenez et al., 2018). Additionally, cold exposure increases the production of reactive oxygen species (ROS). As a reaction to this the antioxidant system is upregulated to prevent oxidative stress (Saltykova, 2019).

As the immune system is complex, the interaction between nutrition and the immune system is as complicated. Nutrition can influence the functioning of the immune system in multiple ways, including the innate immune system, adaptive immune system, and microbiome (Pecora et al., 2020). Additionally, the immune system can also influence the need for and response to nutrition and its metabolism. First of all, it is important that the nutritional status of military personnel is adequate before CWOs, meaning that deficiencies should be prevented. Specifically, under-consumed nutrients which pose a public health concern (so called shortfall nutrients e.g., calcium, potassium, dietary fiber and vitamin D for the U.S. population), should be verified (NIH, 2015; Lutz et al., 2019). Second, there are some nutrients which may be feasible to use as an acute intervention during CWOs.

Nutritional status is closely linked to micronutrient deficiencies. Several micronutrients, including vitamin A, D, C, E B6 and B12, folate, zinc, iron, copper and selenium, are essential for the immune system to function properly (Maggini et al., 2018; Gombart et al., 2020). Vitamin D and A play a crucial role in the immune response (Mora et al., 2008), as these vitamins affect the immune response in a highly specific manner. Vitamin D can be metabolized by immune cells and affects functions of monocytes, macrophages, dendritic cells, T- and B-cells. The specificity of vitamin A metabolites is demonstrated by its effect on T-cell proliferation, mediated by ameliorating IL-2 secretion. Other vitamins, such as C and E, have a less specific effect, but play an important role as antioxidant. The role of minerals in immune function is often as cofactor of metalloenzymes. For instance, zinc acts as cofactor for the enzyme terminal deoxynucleotidyl transferase, which is essential for the replication and functioning of immature T-cells. To support overall health, a minimum dietary intake of 20 mg/day of zinc is recommended (McClung and Scrimgeour, 2005). Zinc is present in high amounts in beef and oysters (Ross et al., 2012). However, in the US breakfast cereals are often fortified with zinc, this could be a strategy for the rations. In addition, iron has an important role in physical, cognitive and immune functions (Beard, 2001) but it is also essential in the body’s ability to regulate temperature when cold stressed (Brigham et al., 1996). So, adequate levels of vitamins and minerals are needed for the immune system to work properly, certainly among military personnel because of their increased risk of attracting an infection as a result of challenging working demands and environments (Hatch-McChesney and Smith, 2023).

Nevertheless, it is known that in military populations, as in most populations, micronutrient deficiencies are present. For example, a study conducted with US military service members showed that iron deficiency was low (104 cases per 100.000 person-years) but present and increased (by 108% in men and 177% in women) in military populations in recent years (Knapik et al., 2021a). In addition, in another study conducted in female military recruits iron deficiency was ∼30% (Israeli et al., 2008). Another study reported micronutrient deficiency present in military personnel is vitamin D deficiency (Fogleman et al., 2022). Supplementation of these micronutrients, as a more acute intervention, has been shown to be effective and should therefore be considered (Fogleman et al., 2022; McClung et al., 2006), especially for deployability during demanding and stressful environments like CWO. For military personnel, 1,500–2000 IU/day is advised as routine supplementation of vitamin D, or up to 4000 IU/day when dietary intake is low (Fogleman et al., 2022). For iron, the MDRI is 8 and 18 mg/d for men and women, respectively (MCO 10110.49, 2017). An alternative could be to fortify breakfast cereals with iron as is common in the US, Canada and many other countries where wheat and other flours are fortified with iron (Gera et al., 2012).

Besides micronutrients, also specific macronutrients affect nutritional status and the immune function. Polyunsaturated fatty acids (PUFAs) have a significant impact on the immune system, influencing immune responses and immune cell functioning. PUFAs, particularly omega-3 PUFAs, have been shown to have immune regulatory functions and can modulate immune cell activity (Radzikowska et al., 2019; Al-Khalaifah, 2020; Duah et al., 2023). These fatty acids can affect the composition and fluidity of cell membranes and act through specific receptors, influencing the effector and regulatory functions of immune cells (Kumar et al., 2019). Studies have demonstrated that PUFAs play a role in immune development, immune maturation, and immune homeostasis, with clinical implications for allergic, autoimmune, and metabolic diseases. The bioactive lipid mediators derived from PUFAs, such as eicosapentaenoic acid (EPA) and docosahexaenoic acid (DHA), have been shown to modulate immune cell signaling and reduce systemic inflammation. Overall, PUFAs have a significant effect on the immune system and can play a role in immune regulation and disease prevention. In the MDRI, only specific recommendations for linoleic acid (18 g/d and 12 g/d for men and women, respectively) and α-linolenic acid (1.6 g/d and 1.1 g/d for men and women, respectively) are mentioned (MCO 10110.49, 2017). Recommended intakes of other PUFAs should be further investigated. Long term consumption of a functional high quality diet including fatty fish or a supplement before and during CWO should be investigated more thoroughly to elaborate on the role of PUFA’s for immune functioning.

Besides vitamins and minerals, other dietary compounds such as Echinacea purpurea and probiotics, are associated with the immune system. These dietary compounds could potentially be used as a more acute intervention during CWOs. However, direct evidence of positive effects on immune function of these dietary immunostimulants in physically active populations are often lacking. For example, the scientific evidence on the immunomodulatory effect of Echinacea purpurea is modest and has mixed outcomes which should be interpreted with caution due to the differences in preparations of Echinacea that were used and the lack of characterization of the test product in many articles. In general, there is only a trend in prevention of colds (Woelkart et al., 2008). In cell cultures, Echinacea purpurea has been shown to stimulate macrophages to produce pro-inflammatory cytokines and reactive oxygen and nitrogen species, which can help in the defense against infections (Vieira et al., 2022).

Also, probiotics may have an immunostimulatory effect, via interaction with intestinal immune cells (Mazziotta et al., 2023; Ashraf and Shah, 2014). One study investigated the effect of probiotics on respiratory tract infections during military training (Tiollier et al., 2007). Even though there were no differences in the number of respiratory tract infections compared to placebo, there was a difference in spread within the respiratory tract, hinting towards a beneficial role of probiotics. More research is needed to confirm the potential beneficial effects of probiotics in CWOs.

Together, this shows the importance of micronutrients and an adequate nutritional status in immune function, also in CWOs. On the one hand, (long existing) deficiencies in nutritional status can impair immune function. On the other hand, some micronutrients could be supplemented to support the immune function during demanding activities like CWOs. Supplementation should result in intakes to approximate the military dietary reference intake (MDRI) (MCO 10110.49, 2017). For some micronutrients, including vitamin D, A, C and E, zinc and iron, the evidence for supplementation is prevailing, for others (e.g., Echinacea purpurea and probiotics) more research is needed to elicit its effect, especially during CWOs.

### 2.2 Vascular response: vasoconstriction versus vasodilation

The human body typically tries to maintain a relatively constant core temperature of about 37°C, even during extreme conditions such as cold exposure. As blood transfers heat throughout the body, the body uses peripheral vasoconstriction to maintain a constant core temperature and prevent heat loss. However, this leads to a decrease in the temperature of the extremities by reducing local blood flow, which can eventually result in cold weather injuries (CWIs). After a few minutes of cold exposure, blood vessels in the extremities show repeated phases of vasodilation, resulting in increased blood flow and subsequent increased heat flow towards the extremities. This is called cold-induced vasodilation (CIVD) (Daanen, 2003). Theoretically the prevention of CWIs can potentially be mediated by CIVD. Yet, evidence investigating whether CIVD is a predisposing factor for the development for CWIs is equivocal (Daanen and Van Der Struijs, 2005; Sullivan-Kwantes et al., 2019).

Dietary intake can contribute to vasodilation in several ways. First, due to dietary-induced thermogenesis (DIT) core temperature could be increased by up to ∼0.3°C and total energy expenditure could be increased by 10% post-prandially, leading to an increased blood flow due to vasodilation (Hirai et al., 1991; Takano and Kotani, 1989; Nielsen, 1987). When comparing the CIVD index – the increase in skin temperature following ice water immersion – pre-prandial to 30 and 90 min post-prandial, 69% and 50% higher skin temperatures were found, respectively, indicating a large vasodilatory effect (Takano and Kotani, 1989). Additionally, oxygen consumption increased with 15% post-prandial compared to pre-prandial. DIT might thus accelerate CIVD. Therefore, micronutrients related to DIT - described in the next chapter - may also have the potential to increase vasodilation, and accordingly decrease peripheral CWIs. Furthermore, the risk of CWIs may be mitigated via a direct effect on vasodilation, which will be discussed in the following paragraphs.

Nitrate or nitric oxide is a micronutrient known for its acute vasodilatory properties. The underlying mechanism by which nitrate influences vasodilation remains unclear, but likely involves direct stimulation of cutaneous vasodilation or sympatho-inhibition (Daanen, 2003). A well-known example of food high in nitrate is beetroot and previous studies indeed found that intake of beetroot juice acutely increased cutaneous blood flow in healthy individuals and people with Raynaud’s phenomenon (Levitt et al., 2015; Keen et al., 2015; Hobbs et al., 2013; Shepherd et al., 2019; Wakabayashi et al., 2023). Wickham and colleagues were not able to show the vasodilatory response during cold water immersion following beetroot juice intake (Wickham et al., 2021), which is possibly explained by the preceding warm water immersion (35°C for 10 min). In summary, most evidence regarding nitrate points towards a stimulating effect on cutaneous vasodilation, using dosages ranging from ∼5 to 12.9 mmol nitrate or nitric oxide. On a practical note, consuming beetroot juice during CWOs could be challenging in terms of logistics as it might freeze when temperature drops below 0°C. Alternatives like beetroot powder should therefore be considered for military purposes in CWOs.

There are more micronutrients acting via the same suggested pathways as nitric oxide. L-citrulline and L-arginine are micronutrients that may improve the vasodilatory response in the cold (Schwedhelm et al., 2008). The vasodilatory effect of L-citrulline and L-arginine has thus far only been shown in the core of the human body (i.e., brachial artery). Nevertheless, supplementation of these components offers an interesting perspective in CWO. Garlic has been shown to affect this nitric oxide pathway via stimulation of hydrogen sulfide (H_2_S) production, resulting in vasodilation (Ried and Fakler, 2014). An additional effect of garlic is the blockage of angiotensin-II production, via inhibiting angiotensin-converting enzyme (ACE) activity, preventing vasoconstriction (Shouk et al., 2014). Dosages in these studies ranged from 600–900 mg, which is less than a clove of garlic (≈2 g), indicating its feasibility for use during CWOs (Rao et al., 2011). Purkayastha et al., (1999) (Purkayastha et al., 1999) studied whether supplementation of vitamin C (500 mg/d), vitamin E (400 mg/d), or a combination of the two (500 and 400 mg/d) resulted in more beneficial CIVD responses under a local cold stimulus, compared to a placebo group. All three groups with supplementation showed better CIVD responses than the placebo group. Vitamin C supplementation (500 mg/d) improved peripheral blood flow most effectively.

Current anecdotical practices for mitigating CWIs include alcohol usage and/or drinking hot beverages. Even though these strategies are known for an acute feeling of warmth, they could lead to heat loss in the long term (Graham and Lougheed, 1985). A more promising strategy may be supplementation of specific micronutrients, as multiple micronutrients seem capable to improve vasodilation in CWOs, possibly (partly) counteracting the negative direct effect of cold exposure itself on peripheral blood circulation. Although direct evidence in military personnel is currently lacking, these micronutrients (i.e., vitamin C, nitric oxide, L-citrulline, L-arginine, and garlic) are highly interesting to investigate and possibly apply in CWOs in military personnel.

### 2.3 Dietary-induced thermogenesis

DIT, also known as the thermic effect of food, refers to the increase in energy expenditure that occurs after consuming a meal. Normally the DIT (assuming an average energy intake of ∼2,500 kcal) is ∼6–20 W (∼5–15% of daily energy expenditure) above basal metabolic rate of ∼60–100 W (Westerterp, 2004). In CWOs the energy expenditure is typically high and the nutritional intake of military personnel is insufficient on average, resulting in caloric deficits of up to 70% of total energy needs of 5,000–7,000 kcal per day (Margolis and Pasiakos, 2023; Margolis et al., 2014; Margolis et al., 2016). In military context, this energy deficit hampers and lowers the DIT as well. Assuming a high energy need and depending on the exact energy intake and deficit, this could cause a lower heat production of the body of ∼10–20 W. Even though this effect is very small (i.e., think of an activity equivalent of fidgeting), it could become relevant during forced static situations in military CWOs. Acute nutritional interventions increasing DIT could therefore be of interest in CWOs.

Not all nutrients contribute equally to DIT. The three main macronutrients—carbohydrates, proteins, and fats—each have different effects on thermogenesis. The DIT of carbohydrates is estimated to be around 5%–10% of caloric intake, while for fats and protein this is around 0%–5% and 20%–30%, respectively (Whitney and Rolfes, 2013). Therefore, to enhance DIT, including a higher proportion of protein in the diet may be beneficial, while maintaining a balanced diet that includes all three macronutrients for overall health. Increasing protein content could be beneficial as protein content of rations for CWO do not always meet the guidelines. Effects may differ between individuals and context, as factors such as meal size, frequency, and individual metabolic rate can also influence the overall thermic effect of a diet (Mitchell et al., 1946). Ready to eat foods, such as high-energy high protein bars could be used (Tanskanen et al., 2012).

While micronutrients do not contribute to DIT in the same way as macronutrients, certain micronutrients can indirectly influence metabolism and as such enhance thermogenesis. A compound that is well-known for its contribution to DIT is capsaicin, a capsaicinoid present in chili peppers and often used for hot sauces (Dulloo, 2011; Bonet et al., 2017; Tremblay et al., 2016; Whiting et al., 2012). Like caffeine, capsaicin (administered as 1 g of red pepper) stimulates adrenaline release in humans, temporarily increasing metabolism by ∼10W of extra heat production and increasing core temperature with 0.02°C over 270 min compared to a control condition (Ludy and Mattes, 2011). The increasing effect of capsaicin ingestion on metabolic heat production is rather direct, making it an interesting micronutrient in the framework of military CWOs. On the other hand, capsaicin also reduces appetite (Whiting et al., 2012), which may not be beneficial for CWOs. Moreover, research suggests that the body can adapt to the thermogenic effect of capsaicin, reducing the effects when the component is used for longer periods of time (i.e., for regular users with a preferred quantity of 1.8 g red pepper per meal, compared to non-users with 0.3 g per meal) (Ludy and Mattes, 2011). Therefore, considering its limited effect on core temperature, the reduction in appetite and the adaptation to capsaicin, this micronutrient might not be suitable for CWIs prevention. However, it might be useful in unanticipated acute and critical situations where no other preventive measures are available. Capsaicin could also be applied topically, which could affect thermal sensations and consequently cause a local warmer feeling (Callsen et al., 2008). In the military, it is not recommended to blunt thermal sensations as behavior may change inappropriately after applying a topical like capsaicin. The same principle holds true for cinnamon or cinnamaldehyde (Jiang et al., 2017). Cinnamaldehyde increases both thermogenesis and fat oxidation. Cinnamaldehyde could potentially have advantages over capsaicin supplementation since it is perceived as more tolerable, while enhancing energy expenditure, where capsaicin did not compared to placebo (Michlig et al., 2016). Future research should quantify the thermogenic effects of the above-mentioned spices, including the impact of chronic intake.

Several other nutritional/bioactive compounds have been attributed with thermogenic properties, such as ginger, L-menthol, ephedrine and xanthine, calcium and vitamin D and some minerals and polyphenolic compounds. Ginger increases the synthesis and expression of uncoupling protein 1 (UCP1) by activating the sympathetic nerve system through affecting beta-adrenergic receptors. Hence, ginger enhances thermogenesis via dissipating the protein gradient in oxidative phosphorylation, which reduces ATP production – thus increasing heat production–in brown adipose tissue (BAT) (Bonet et al., 2017; Fujisawa et al., 2005). The effect of ginger on UCP1 has been shown in animal models and in human clinical trials (Bonet et al., 2017). In animals, L-menthol has been shown to activate UCP1 through stimulation of the transient receptor potential melastatin 8 ion-channel (TRPM8) (Bonet et al., 2017; Sanders et al., 2021). This TRPM8 channel is a receptor mainly involved in thermal stimuli in the peripheral nervous system. L-menthol activates these receptors which, ultimately, leads to enhanced thermogenesis, i.e., menthol gives a cold feeling, which results in the production of heat. In humans, oral administration of L-menthol (10 mg/kg) increased metabolic rate by 10% (Valente et al., 2015), and skin administration (10 mg/kg) by 18%. Oral L-menthol comes in capsules to easily consume relatively large amounts. Therefore, during military CWOs, menthol could be a potential solution to stimulate thermogenesis and prevent the development of specific cold weather injury (CWI). Increases in metabolic heat production can also be achieved by a mixture of ephedrine and xanthine (such as caffeine and theophylline). Studies investigating such mixtures in the cold observed increases in CHO and/or lipid oxidation (Dulloo, 2011; Bonet et al., 2017; Carlson and Marriott, 1996). This enhanced thermogenesis was reflected in higher core and skin temperature. The isolated effect of both components did not elucidate the same response in terms of thermoregulatory thermogenesis and cold tolerance. The combination of calcium and vitamin D supplementation during breakfast increased DIT (5.1% vs. 6.8% of energy intake, *p* = 0.01) over consecutive meals (Chan She Ping-Delfos and Soares, 2011). This was the result of an increased fat oxidation rate. Some other minerals, including iodine and selenium, have an indirect effect on DIT, as they are part of hormones that influence energy metabolism. Also, iron and zinc are indirectly involved in DIT as they are required in numerous metabolic processes (Rosenzweig and Volpe, 1999; Severo et al., 2019). Polyphenolic compounds, such as in green tea extract and extra virgin olive oil, most likely increase noradrenaline-induced thermogenesis and fat oxidation (Dulloo, 2011; Dulloo et al., 1999), increasing heat production. Therefore, it seems that a variety of nutritional/bioactive compounds like ginger, caffeine and polyphenolic compounds are capable of stimulating thermogenesis. However, their effect on the development of CWIs remains unclear.

It seems worth to investigate the effectiveness of ingestion of abovementioned micronutrients and nutritional compounds (ginger, caffeine, polyphenolic compounds) in military CWOs. As most of the described nutrients are required in relatively small amounts to induce a thermogenic effect, and combinations of these nutrients could be used, adding these nutrients to (specific products) in the rations could prove a practically feasible intervention. Future research should demonstrate which products are most suited, for instance by fortifying those products in the ration that are already frequently consumed during CWOs. Also, in future studies it should be demonstrated whether the ingestion of those spices and micronutrients during CWOs reduces the risks of developing CWIs. Regarding military field work, if the ingestion of those micronutrients and nutritional compounds lowers the risk of developing CWIs, adding spices and/or micronutrients to the rations could be a practical solution for a direct thermogenic effect in (anticipated) critical situations.

## 3 Cognitive mechanisms

### 3.1 Cognitive function

Cold exposure has been found to have negative effects on cognitive function (Taylor et al., 2016), particularly on attention, processing speed, executive function, and memory (Falla et al., 2021). The decrease in cognitive function might be explained by distraction or arousal due to the effects of cold exposure (Teichner, 1958; Enander, 1987; Ellis, 1982).

Some nutritional compounds have been shown to positively affect cognitive function, including caffeine and tyrosine. Caffeine is recognized as an ergogenic substance that improves both physical and cognitive function. In military context, caffeine has been extensively studied, demonstrating its benefical effects on attention, vigilance, reaction time, problem soliving, and reasoning with doses ranging from 100–800 mg (Chaudhary et al., 2021; Crawford et al., 2017; Lieberman et al., 2002). In addition, Lieberman et al. showed that caffeine improved cognitive function 1 h after administration, specifically visual vigilance, choice reaction time, repeated acquisition, self-reported fatigue and sleepiness, during U.S. Navy SEAL training enduring severe environmental and operational stress, including cold (Lieberman et al., 2002). However, not much research has been done on the effect of caffeine on cognitive function specifically in cold environments in a militairy context. The 2001 Institute of Medicine report concluded that additional research was needed on the appropriatness of caffeine supplementation during various military situations (IOW 2001). To date, even though a body of evidence exists on the efficacy of caffeine supplementation in moderate environments and situations, the conclusion of the Institute of Medicine still seems to stand for CWOs. Regarding caffeine intake, it is known that caffeine consumption via coffee is typically high amongst military personnel (Knapik et al., 2022a). It should be determined what amount of caffeine would be needed on top of that to achieve similar cognitive benefits. Caffeine administration through gum or capsules rather than in coffee (26 mg caffeine/100 g instand coffee (USDA, 2019)) may be more suitable to consume the required amounts.

An important amino acid for proper cognitive function is tyrosine, which is a precursor of catecholamines dopamine, epinephrine, and norepinephrine (Mahoney et al., 2007). Increasing tyrosine levels could increase brain catecholamine synthesis and thereby lead to improved cognitive performance and mood, especially under stressful conditions during which brain catecholamine levels may be reduced (Hase et al., 2015). Supplementation with 2 g/d tyrosine for 5 days increased the performance on a memory and tracking task during operational circumstances of a combat training course (Deijen et al., 1999). A similar effect was found after cooling of the body, which resulted in a decline in cognitive function, but the decline in working memory was counteracted when tyrosine (300 mg/kg body weight) was supplemented before cold exposure (Mahoney et al., 2007; O’Brien et al., 2007). These studies confirm that cold exposure decreases cognitive function and that this effect can potentially be counteracted with tyrosine supplementation (∼2 g/d, considering that ∼2 h after administration maximum levels of tyrosine in the blood were reached).

In terms of nutritional benefits on cognitive function, phytochemicals may also be promising. Phytochemicals are plant compounds which can be divided into polyphenols, terpenoids, nitrogen- and sulfur-containing compounds (Fraga et al., 2019). In particular polyphenols, especially flavonoids, are well-studied because of their potential health benefits. Previously, polyphenols were mostly known as strong antioxidants. However, as knowledge about polyphenols is expanding, it becomes clear that they also positively affect multiple other processes, including neuronal function (Vauzour, 2012). The systematic review of Teo et al. (2017a) aimed to develop recommendations for military personnel by assessing the evidence of phytochemicals on cognitive function in a healthy population. Generally, no significant changes in cognitive function are found for isoflavones and cocoa polyphenols. Another systematic review found positive effects on cognitive performance and increased cerebral blood flow and oxygenation following consumption of cocoa polyphenols in young adults (Martín et al., 2020). Scholey et al. (2010) showed cognitive improvements, including reduced mental fatigue ratings and improved working memory, after cocoa flavanol (520 mg) consumption. Given the lack of consensus in current literature, further research is needed to draw conclusions, especially for the translation to CWOs.

Two other compounds, nitrate and creatine, which are known for their beneficial effect on physical performance, might also positively influence cognitive function. Nitrate modulates cerebral blood-flow (Wightman et al., 2015) which may result in improved cognitive function, especially reaction time, during a prolonged intermittent sprint test (Thompson et al., 2015). If nitrate supplementation may improve cognitive function of military personnel in CWOs remains to be elucidated.

Creatine is known for its positive effect on muscle strength and recovery from intense exercise (Branch, 2003). Creatine supplementation positively affects cognitive function as well. Rae et al. (2003) showed that 5 g/d creatine for 6 weeks improved working memory and intelligence. Watanabe et al. (2002) showed that 8 g/d for 5 days reduced mental fatigue during repeated tasks. On the other hand, Rawson et al. (2008) did not show improvements in cognitive function with 0.03 g/kg/d creatine supplementation for 6 weeks. This might indicate that the dose should be higher than 0.03 g/kg/d (i.e. 2.4 g/day for a body weight of 80 kg), which is in accordance with the current advice of a maintenance load – without a preceding loading phase – of 3–5 g/d for a minimum of 4 weeks for improving muscle strength and performance (Naderi et al., 2016). Additional research on appropriate dosing needs to be done to establish these findings in CWOs as well.

Long term higher intake of omega-3 fatty acids is associated with a better cognitive function in clinical populations (Mazereeuw et al., 2012). However, a systematic review looking into omega-3 fatty acids to optimize military mission-readiness did not show an effect of omega-3 fatty acids on cognitive performance in healthy persons (Teo et al., 2017b). This was confirmed in military context, showing no effect of omega-3 supplementation (2.5 g/d) on cognitive performance (Dretsch et al., 2014). Likewise, the study of (Marriott et al., 2021) did not show an effect of omega-3 (2.3 g/d for 20 weeks) on cognitive performance in young military officers. However, in this study, compliance was low as blood spot samples did not show expected elevations of omega-3. Even though evidence is scarce, military personnel could potentially benefit from long-term omega-3 supplementation, even more because consumption of omega-3 fatty acids are generally low (Muldoon et al., 2014). This endorses the idea that a good nutritional health status, hence the absence of nutritional deficits, is important before and during CWOs to maintain cognitive function.

In conclusion, for some micronutrients, especially tyrosine and to a lesser extent caffeine, there is emerging evidence to support its use in CWOs as a cognitive performance enhancer. There seems to be potential in other micronutrients including nitrate, creatine, and omega-3 fatty acids in healthy populations, but their potential cognitive benefits in military personnel during cold exposure should be established.

### 3.2 Psychology and cognitive wellbeing

Not only cognitive function, but also psychological factors and cognitive wellbeing are affected by cold exposure (Lieberman et al., 2009; Palinkas and Suedfeld, 2008). Even though this has not been studied much during CWOs in military personnel, the “will to survive” has widely been accepted as important to survive in extreme conditions (Barwood et al., 2006). This “will to survive” often refers to several abilities, often related to tolerating or suppressing unpleasant sensations (Barwood et al., 2006) and a mental preparation to be “comfortably cold” (Sullivan-Kwantes et al., 2021). Mental preparedness to CWOs necessitates a person to have several psychological characteristics, including a high motivation and a low susceptibility to anxiety (Palinkas and Suedfeld, 2008). Psychological symptoms experienced by people on polar expeditions include a depressed mood, and increased anger, irritability, and anxiety (Palinkas and Suedfeld, 2008). Therefore, dietary interventions to mitigate those psychological symptoms may improve (long-term) deployability of military personnel in CWOs.

Regarding dietary fibers, there is quite some evidence on its beneficial health effects (Dreher, 2018). Furthermore, observational studies have shown that dietary fibers have a beneficial effect on psychological wellbeing and lower the risk of depression. This link between dietary fibers and depression is thought to be due to the effect of dietary fibers on inflammation (Swann et al., 2020). Even though no studies have been conducted during CWOs, it is known that cold exposure can induce changes in the gut microbiota. Dietary fibers are important for this, as they affect the microbiota-gut-brain axis (Zhou et al., 2024). This indicates that dietary fibers may be beneficial during CWOs, but may also be of interest as a long-term strategy in improving psychological wellbeing and lowering the risk of depression, especially since it is known that dietary fiber intake is low in military personnel. Adding products high in dietary fiber, e.g., whole grain bread, vegetables and legumes, or supplements would be useful to reach the MDRI of 34 g and 28 g per day for men and women, respectively (MCO 10110.49, 2017), however, more research is needed.

Besides its effect on cognitive function, tyrosine also affects mood (Banderet and Lieberman, 1989), as 100 mg/kg body weight tyrosine could decrease adverse moods during cold and hypoxia exposure. However, as this study was performed in a non-military context, this effect of tyrosine needs to be confirmed during CWOs in a military context.

To conclude, for cognitive wellbeing, dietary fibers are the most promising nutrient, although this needs to be confirmed in the context of CWOs, by studying the effects of dietary fibers in modulating the gut microbiota during CWOs, or by exploring the effects of a longer-term increase in fiber intake on psychological wellbeing.

## 4 Recommendations and summary

**FIGURE 1:**
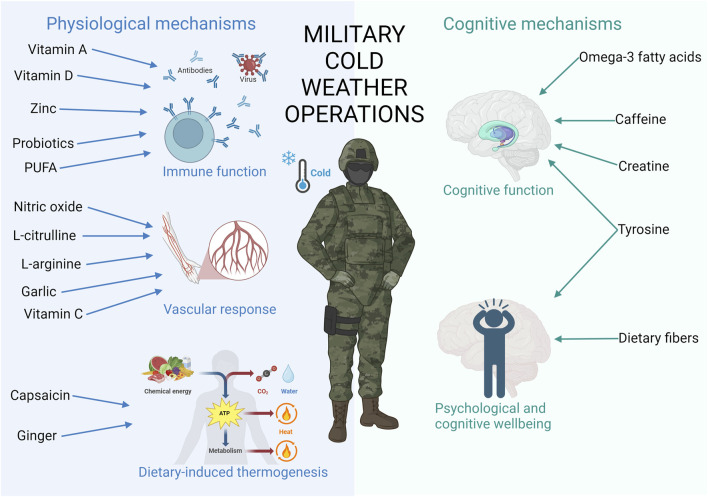
This figure contains important physiological and cognitive mechanisms affected by cold, leading to detrimental military performance during CWO. In addition, the most promising nutrients improving those mechanisms are added. Created in https://BioRender.com.

The aim of this narrative review was to provide an overview of the potential effects of nutritional strategies on several physiological and cognitive mechanisms to enhance military performance in CWOs (Figure 1). Additionally, the aim was to offer nutritional recommendations for military performance enhancement in CWOs.

As discussed, exposure to (extreme) cold environments influences various physiological mechanisms and could have a detrimental effect on military performance (and safety). It is imperative for military personnel to have an adequate nutritional status before leaving for CWOs, as this optimizes bodily functions and enables a well-functioning immune system. A well-functioning immune system is essential to withstand the hardships of CWO. Most vitamins and minerals are essential for immune function, however, focusing on vitamin D, A and zinc intake seems to be most effective. PUFAs are also pivotal for the immune system, and the beneficial effect of probiotics seems promising, although this should be further investigated.

In addition, the response of the vascular system to cold exposure is one of most important ones to survive and last in cold environments. A proper function and regulation of the vascular system will prevent both central (hypothermia) and peripheral (e.g., frostbites) CWIs, and can increase military performance. Besides producing heat within the human body, DIT can significantly improve the CIVD response, possibly lowering the risk of CWIs of the extremities. Several micronutrients have been shown to be capable of positively influencing vascular response, like vitamin C, nitric oxide, L-citrulline and L-arginine, and garlic. Although direct evidence in military personnel is currently lacking, these nutrients are highly interesting to test and possibly apply in CWOs.

Commonly, the DIT is lowered during CWOs compared to normal conditions, because of the relatively low caloric intake compared to high caloric demand (Margolis et al., 2014). Either increasing caloric intake, or increasing DIT via micronutrients and bioactive compounds, ideally both, may increase DIT and subsequently decrease risk of CWIs during specific tasks when insufficient heat is generated via physical activity to preserve core temperature. Capsaicin and ginger are two micronutrients which could increase DIT. Adding these spices to rations could be relatively easy and could be a practical solution for a direct thermogenic effect in (anticipated) critical situations.

In terms of micronutrients beneficially influencing cognitive function and wellbeing during CWOs, most evidence is present for caffeine, tyrosine, and dietary fibers. Although, there are some other promising nutrients that could be investigated for their use in CWO, including creatine and nitrate. Omega-3 fatty acids are well-known for enhancing cognitive function in the long term. However, no effects are found in studies with military personnel or in the cold. Addition of omega-3 fatty acids to rations could be relevant as energy intake is generally low and its addition could be beneficial.

It is important to realize that micronutrient supplementation is not the only and certainly not the most effective mitigation strategy in CWOs. For example, intense exercise will generate much more heat production than a micronutrient intervention increasing DIT. However, some large impact interventions (exercise and adding clothing insulation) cannot be undertaken at all moments due to the nature of military combat. Other interventions like micronutrient supplementation may be applicable in certain scenarios (e.g., observational tasks) in CWOs. In addition, as innovations and new capabilities are getting more important and geopolitical changes are forcing us to investigate all possible wins, it is important to add micronutrients to the CWOs ‘toolbox’ as well and apply such strategies were possible.

It remains to be established if the potential to improve vasodilation may actually decrease risk of CWIs. From a practical point of view during actual CWOs, where it matters most, it will be challenging to study this for several reasons, e.g., due to a lack of controlling environmental conditions. A solution may be to include large groups of military personnel in those field-based studies, or to test the interventions in controlled, simulated environments like climate chambers. Therefore, intervention studies, both controlled and in a field-setting, investigating both effective administration as well as (a mix of) specific micronutrients over a longer period, would provide essential information for military personnel during CWOs.

Also, it is known that energy intake is lowered during CWOs (Margolis and Pasiakos, 2023). Therefore, nutritional interventions should aim for energy balance, for example by introducing multiple food breaks during CWOs (Charlot et al., 2021). Besides energy intake, improving overall diet quality of military personnel, including shortfall nutrients, should be prioritized to prevent deficiencies and improve overall performance. However, research shows that compliance with supplements among military personnel may be low (Gofrit et al., 2023). To improve compliance with a nutritional intervention, nutrients such as iron, zinc, calcium vitamin D, PUFA’s and fibers could also be added to high-energy high-protein snacks in the rations. Such a strategy could potentially tackle both the deficiency in energy intake and shortfall in micronutrient intake. In addition, from a practical point of view, administration of abovementioned micronutrients in a CWOs context may not be as easy as during moderate temperatures because liquids (e.g., most common way of administration of nitric oxide is via beetroot juice) may freeze during CWOs. To actually apply interventions with micronutrients to enhance vascular response this issue needs to be tackled. For long-term nutritional strategies, supplements may prove more feasible, as research shows dietary supplement use is quite common amongst soldiers (Knapik et al., 2021b; Casey et al., 2014). This would require proper nutrition education to explain the benefit to military personnel and to avoid exceeding the recommended limits of nutrients and potential adverse effects, due to taking combinations of several products (Knapik et al., 2022b). Another difficulty doing research in military practice is to include sufficient military participants to allow for appropriate statistical power. Availability of soldiers for research is often scarce due to the nature of military combat and planning changes whenever geopolitical hazards increase or decrease. Having sufficiently powered results in the specific setting is essential to allow for translatable knowledge.

To summarize, although some specific recommendations can be made, it is clear that a lot of knowledge is still missing around the efficacy and use of nutrients in CWOs. Although a reasonable body of knowledge exists on how either (extreme) cold exposure or micronutrients influence the abovementioned physiological and cognitive mechanisms, more emphasis should be placed on integrating this knowledge and conducting research in a relevant setting. Additionally, it is of interest to explore which combinations of nutrients will be most advantageous.

## References

[B1] Al-Khalaifah H. (2020). Modulatory effect of dietary polyunsaturated fatty acids on immunity, represented by phagocytic activity. Front. Vet. Sci. 7 (September), 569939–570021. 10.3389/fvets.2020.569939 33195556 PMC7536543

[B2] Ashraf R. Shah N. P. (2014). Immune system stimulation by probiotic microorganisms. Crit. Rev. Food Sci. Nutr. 54 (7), 938–956. 10.1080/10408398.2011.619671 24499072

[B3] Banderet L. E. Lieberman H. R. (1989). Treatment with tyrosine, a neurotransmitter precursor, reduces environmental stress in humans. Brain Res. Bull. 22 (4), 759–762. 10.1016/0361-9230(89)90096-8 2736402

[B4] Barwood M. J. Dalzell J. Datta A. K. Thelwell R. C. Tipton M. J. (2006). Breath-hold performance during cold water immersion: effects of psychological skills training. Aviat. Sp. Environ. Med. 77 (11), 1136–1142. 17086766

[B5] Beard J. L. (2001). Iron biology in immune function, muscle metabolism and neuronal functioning. J. Nutr. 131 (2S-2), 568–580. 10.1093/jn/131.2.568S 11160590

[B6] Bonet M. L. Mercader J. Palou A. (2017). A nutritional perspective on UCP1-dependent thermogenesis. Biochimie 134, 99–117. 10.1016/j.biochi.2016.12.014 28057582

[B7] Branch J. D. (2003). Effect of creatine supplementation on body composition and performance: a meta-analysis. Int. J. Sport Nutr. Exerc Metab. 13 (2), 198–226. 10.1123/ijsnem.13.2.198 12945830

[B8] Brigham D. Beard J. Tobin B. (1996). Iron and thermoregulation: a review. Crit. Rev. Food Sci. Nutr. 36 (8), 747–763. 10.1080/10408399609527748 8989508

[B9] Callsen M. G. Moller A. T. Sorensen K. Jensen T. S. Finnerup N. B. (2008). Cold hyposensitivity after topical application of capsaicin in humans. Exp. brain Res. 191 (4), 447–452. 10.1007/s00221-008-1535-1 18709364

[B10] Carlson S. J. Marriott B. M. (1996). Nutritional needs in cold and high-altitude environments: applications for military personnel in field operations. Washington DC, USA: National Academies Press. 25121290

[B11] Casey A. Hughes J. Izard R. M. Greeves J. P. (2014). Supplement use by UK-based British Army soldiers in training. Br. J. Nutr. 112 (7), 1175–1184. 10.1017/S0007114514001597 25119518 PMC4189117

[B12] Castellani J. W. Spitz M. G. Karis A. J. Martini S. Young A. J. Margolis L. M. (2017). Cardiovascular and thermal strain during 3-4 days of a metabolically demanding cold-weather military operation. Extrem Physiol. Med. 6 (1), 2–13. 10.1186/s13728-017-0056-6 28878888 PMC5586032

[B13] Castellani J. W. Young A. Ducharme M. Giesbrecht G. Glickman E. Sallis R. (2006). American College of Sports Medicine position stand: prevention of cold injuries during exercise. Med. Sci. Sport Exerc 38 (11), 2012–2029. 10.1249/01.mss.0000241641.75101.64 17095937

[B14] Chan She Ping-Delfos W. Soares M. (2011). Diet induced thermogenesis, fat oxidation and food intake following sequential meals: influence of calcium and vitamin D. Clin. Nutr. 30 (3), 376–383. 10.1016/j.clnu.2010.11.006 21276644

[B15] Charlot K. Chapelot D. Siracusa J. Lavoué C. Colin P. Oustric P. (2021). An augmented food strategy leads to complete energy compensation during a 15-day military training expedition in the cold. Physiol. Rep. 9 (11), 145911–e14620. 10.14814/phy2.14591 PMC8165736 34057319

[B16] Chaudhary N. S. Taylor B. V. Grandner M. A. Troxel W. M. Chakravorty S. (2021). The effects of caffeinated products on sleep and functioning in the military population: a focused review. Pharmacol. Biochem. Behav. 206 (April), 173206. 10.1016/j.pbb.2021.173206 34000324 PMC8487254

[B17] Crawford C. Teo L. Lafferty L. Drake A. Bingham J. J. Gallon M. D. (2017). Caffeine to optimize cognitive function for military mission-readiness: a systematic review and recommendations for the field. Nutr. Rev. 75, 17–35. 10.1093/nutrit/nux007 28969341

[B18] Daanen H. A. M. (2003). Finger cold-induced vasodilation: a review. Eur. J. Appl. Physiol. 89 (5), 411–426. 10.1007/s00421-003-0818-2 12712346

[B19] Daanen H. A. M. Van Der Struijs N. R. (2005). Resistance index of frostbite as a predictor of cold injury in arctic operations. Aviat. Sp. Environ. Med. 76 (12), 1119–1122. 16370261

[B20] Deijen J. B. Wientjes C. J. E. Vullinghs H. F. M. Cloin P. A. Langefeld J. J. (1999). Tyrosine improves cognitive performance and reduces blood pressure in cadets after one week of a combat training course. Brain Res. Bull. 48 (2), 203–209. 10.1016/s0361-9230(98)00163-4 10230711

[B21] Dreher M. L. (2018). Whole fruits and fruit fiber emerging health effects. Nutrients 10 (12), 1833. 10.3390/nu10121833 30487459 PMC6315720

[B22] Dretsch M. N. Johnston D. Bradley R. S. MacRae H. Deuster P. A. Harris W. S. (2014). Effects of omega-3 fatty acid supplementation on neurocognitive functioning and mood in deployed U.S. soldiers: a pilot study. Mil. Med. 179 (4), 396–403. 10.7205/MILMED-D-13-00395 24690964

[B23] Duah M. Zhang K. Liang Y. Ayarick V. A. Xu K. Pan B. (2023). Immune regulation of poly unsaturated fatty acids and free fatty acid receptor 4. J. Nutr. Biochem. 112, 109222. 10.1016/j.jnutbio.2022.109222 36402250

[B24] Dulloo A. G. (2011). The search for compounds that stimulate thermogenesis in obesity management: from pharmaceuticals to functional food ingredients. Obes. Rev. 12 (10), 866–883. 10.1111/j.1467-789X.2011.00909.x 21951333

[B25] Dulloo A. G. Duret C. Rohrer D. Girardier L. Mensi N. Fathi M. (1999). Efficacy of a green tea extract rich in catechin polyphenols and caffeine in increasing 24-h energy expenditure and fat oxidation in humans. Am. J. Clin. Nutr. 70 (6), 1040–1045. 10.1093/ajcn/70.6.1040 10584049

[B26] Ellis H. D. (1982). The effects of cold on the performance of serial choice reaction time and various discrete tasks. Hum. Factors 24 (5), 589–598. 10.1177/001872088202400509 7173879

[B27] Enander A. (1987). Effects of moderate cold on performance of psychomotor and cognitive tasks. Ergonomics 30 (10), 1431–1445. 10.1080/00140138708966037 3428250

[B28] Falla M. Micarelli A. Hüfner K. Strapazzon G. (2021). The effect of cold exposure on cognitive performance in healthy adults: a systematic review. Int. J. Environ. Res. Public Health 18 (18), 9725. 10.3390/ijerph18189725 34574649 PMC8470111

[B29] Fogleman S. A. Janney C. Cialdella-Kam L. Flint J. H. (2022). Vitamin D deficiency in the military: it’s time to act. Mil. Med. 187 (5–6), 144–148. 10.1093/milmed/usab402 34626466

[B30] Fraga C. G. Croft K. D. Kennedy D. O. Tomás-Barberán F. A. (2019). The effects of polyphenols and other bioactives on human health. Food Funct. 10 (2), 514–528. 10.1039/c8fo01997e 30746536

[B31] Fujisawa F. Nadamoto T. Fushiki T. (2005). Effect of intake of ginger on peripheral body temperature. J. Jpn. Soc. Nutr. Food Sci. 58 (1), 3–9. 10.4327/jsnfs.58.3

[B32] Gera T. Sachdev H. S. Boy E. (2012). Effect of iron-fortified foods on hematologic and biological outcomes: systematic review of randomized controlled trials. Am. J. Clin. Nutr. 96 (2), 309–324. 10.3945/ajcn.111.031500 22760566

[B33] Gofrit S. G. Ohayon-Cohen S. Tsur A. M. Rabkin V. Shapira M. M. Finestone A. S. (2023). Compliance compromises an interventional study on iron supplementation in female combatants. BMJ Mil. Heal 169 (1), 27–31. 10.1136/jramc-2019-001245 31235618

[B34] Gombart A. F. Pierre A. Maggini S. (2020). A review of micronutrients and the immune system—working in harmony to reduce the risk of infection. Nutrients 12 (1), 236. 10.3390/nu12010236 31963293 PMC7019735

[B35] Graham T. E. Lougheed M. D. (1985). Thermal responses to exercise in the cold: influence of sex differences and alcohol. Hum. Biol. 57 (4), 687–698. 4086023

[B36] Hase A. Jung S. E. Aan H. R. M. (2015). Behavioral and cognitive effects of tyrosine intake in healthy human adults. Pharmacol. Biochem. Behav. 133, 1–6. 10.1016/j.pbb.2015.03.008 25797188

[B37] Hatch-McChesney A. Smith T. J. (2023). Nutrition, immune function, and infectious disease in military personnel: a narrative review. Nutrients 15 (23), 4999. 10.3390/nu15234999 38068857 PMC10708187

[B38] Hirai A. Tanabe M. Shido O. (1991). Applied Physiology subjects to the increase in body temperature during exercise. Eur. J. Appl. Physiol. Occup. Physiol. 62 (3), 221–222. 10.1007/BF00643746 2044530

[B39] Hobbs D. A. Goulding M. G. Nguyen A. Malaver T. Walker C. F. George T. W. (2013). Acute ingestion of beetroot bread increases endothelium-independent vasodilation and lowers diastolic blood pressure in healthy men: a randomized controlled trial. J. Nutr. 143 (9), 1399–1405. 10.3945/jn.113.175778 23884387

[B40] Israeli E. Merkel D. Constantini N. Yanovich R. Evans R. K. Shahar D. (2008). Iron deficiency and the role of nutrition among female military recruits. Med. Sci. Sports Exerc 40 (11 Suppl. l), 685–690. 10.1249/MSS.0b013e31818946ae 18849865

[B41] Jiang J. Emont M. P. Jun H. Qiao X. Liao J. Kim D. (2017). Cinnamaldehyde induces fat cell-autonomous thermogenesis and metabolic reprogramming. Metabolism 77, 58–64. 10.1016/j.metabol.2017.08.006 29046261 PMC5685898

[B42] Keen J. T. Levitt E. L. Hodges G. J. Wong B. J. (2015). Short-term dietary nitrate supplementation augments cutaneous vasodilatation and reduces mean arterial pressure in healthy humans. Microvasc. Res. 98, 48–53. 10.1016/j.mvr.2014.12.002 25554360

[B43] Klous L. Teien H. Hollis S. Levels K. Boonstra A. Sullivan-Kwantes W. (2024). Cold weather operations: preventive strategies in a military context. 00(00):1–20. 10.1080/23328940.2024.2408059 PMC11875484 40041157

[B44] Knapik J. J. Farina E. K. Fulgoni V. L. Lieberman H. R. (2021a). Clinically-diagnosed vitamin deficiencies and disorders in the entire United States military population, 1997–2015. Nutr. J. 20 (1), 55–13. 10.1186/s12937-021-00708-2 34130698 PMC8207601

[B45] Knapik J. J. Steelman R. A. Trone D. W. Farina E. K. Lieberman H. R. (2022a). Prevalence of caffeine consumers, daily caffeine consumption, and factors associated with caffeine use among active duty United States military personnel. Nutr. J. 21 (1), 22–19. 10.1186/s12937-022-00774-0 35421992 PMC9008906

[B46] Knapik J. J. Trone D. W. Steelman R. A. Farina E. K. Lieberman H. R. (2021b). Prevalence of and factors associated with dietary supplement use in a stratified, random sample of US military personnel: the US military dietary supplement use study. J. Nutr. 151 (11), 3495–3506. 10.1093/jn/nxab239 34293133 PMC8562080

[B47] Knapik J. J. Trone D. W. Steelman R. A. Farina E. K. Lieberman H. R. (2022b). Adverse effects associated with multiple categories of dietary supplements: the military dietary supplement use study. J. Acad. Nutr. Diet. 122 (10), 1851–1863. 10.1016/j.jand.2022.01.014 35123127

[B48] Kumar N. G. Contaifer D. Madurantakam P. Carbone S. Price E. T. Tassell B. V. (2019). Dietary bioactive fatty acids as modulators of immune function: implications on human health. Nutrients 11 (12), 2974–3015. 10.3390/nu11122974 31817430 PMC6950193

[B49] Levitt E. L. Keen J. T. Wong B. J. (2015). Augmented reflex cutaneous vasodilatation following short‐term dietary nitrate supplementation in humans. Exp. Physiol. 100 (6), 708–718. 10.1113/EP085061 25826741

[B50] Lieberman H. R. Castellani J. W. Young A. J. (2009). Cognitive function and mood during acute cold stress after extended military training and recovery. Aviat. Sp. Environ. Med. 80 (7), 629–636. 10.3357/asem.2431.2009 19601505

[B51] Lieberman H. R. Tharion W. J. Shukitt-Hale B. Speckman K. L. Tulley R. (2002). Effects of caffeine, sleep loss, and stress on cognitive performance and mood during U.S. Navy SEAL training. Sea-Air-Land. Psychopharmacol. Berl. 164 (3), 250–261. 10.1007/s00213-002-1217-9 12424548

[B52] Ludy M. J. Mattes R. D. (2011). The effects of hedonically acceptable red pepper doses on thermogenesis and appetite. Physiol. Behav. 102 (3–4), 251–258. 10.1016/j.physbeh.2010.11.018 21093467 PMC3022968

[B53] Lutz L. J. Gaffney-Stomberg E. Karl J. P. Hughes J. M. Guerriere K. I. McClung J. P. (2019). Dietary intake in relation to military dietary reference values during Army basic combat training; A multi-center, cross-sectional study. Mil. Med. 184 (3–4), E223–E230. 10.1093/milmed/usy153 29982620

[B54] Maggini S. Pierre A. Calder P. C. (2018). Immune function and micronutrient requirements change over the life course. Nutrients 10 (10), 1531. 10.3390/nu10101531 30336639 PMC6212925

[B55] Mahoney C. R. Castellani J. Kramer F. M. Young A. Lieberman H. R. (2007). Tyrosine supplementation mitigates working memory decrements during cold exposure. Physiol. Behav. 92 (4), 575–582. 10.1016/j.physbeh.2007.05.003 17585971

[B56] Mäkinen T. M. Juvonen R. Jokelainen J. Harju T. H. Peitso A. Bloigu A. (2009). Cold temperature and low humidity are associated with increased occurrence of respiratory tract infections. Respir. Med. 103 (3), 456–462. 10.1016/j.rmed.2008.09.011 18977127

[B57] Margolis L. M. Murphy N. E. Martini S. Gundersen Y. Castellani J. W. Karl J. P. (2016). Effects of supplemental energy on protein balance during 4-d arctic military training. Med. Sci. Sport Exerc 48 (8), 1604–1612. 10.1249/MSS.0000000000000944 27054679

[B58] Margolis L. M. Murphy N. E. Martini S. Spitz M. G. Thrane I. McGraw S. M. (2014). Effects of winter military training on energy balance, whole-body protein balance, muscle damage, soreness, and physical performance. Appl. Physiol. Nutr. Metab. 39 (12), 1395–1401. 10.1139/apnm-2014-0212 25386980

[B59] Margolis L. M. Pasiakos S. M. (2023). Performance nutrition for cold-weather military operations. Int. J. Circumpolar 82 (1), 2192392. 10.1080/22423982.2023.2192392 PMC10026745 36934427

[B60] Marriott B. P. Turner T. H. Hibbeln J. R. Newman J. C. Pregulman M. Malek A. M. (2021). Impact of fatty acid supplementation on cognitive performance among United States (Us) military officers: the ranger resilience and improved performance on phospholipid-bound omega-3’s (rripp-3) study. Nutrients 13 (6), 1854. 10.3390/nu13061854 34072293 PMC8228047

[B61] Martín M. A. Goya L. Pascual-Teresa S. D. (2020). Effect of cocoa and cocoa products on cognitive performance in young adults. Nutrients 12 (12), 1–14. 10.3390/nu12123691 PMC7760676 33265948

[B62] Mazereeuw G. Lanctôt K. L. Chau S. A. Swardfager W. Herrmann N. (2012). Effects of omega-3 fatty acids on cognitive performance: a meta-analysis. Neurobiol. Aging 33 (7), 1482.e17–e29. 10.1016/j.neurobiolaging.2011.12.014 22305186

[B63] Mazziotta C. Tognon M. Martini F. Torreggiani E. Rotondo J. C. (2023). Probiotics mechanism of action on immune cells and beneficial effects on human health. Cells 12, 184–233. 10.3390/cells12010184 36611977 PMC9818925

[B64] McClung J. P. Marchitelli L. J. Friedl K. E. Young A. J. (2006). Prevalence of iron deficiency and iron deficiency anemia among three populations of female military personnel in the US Army. J. Am. Coll. Nutr. 25 (1), 64–69. 10.1080/07315724.2006.10719516 16522934

[B65] McClung J. P. Martini S. Murphy N. E. Montain S. J. Margolis L. M. Thrane I. (2013). Effects of a 7-day military training exercise on inflammatory biomarkers, serum hepcidin, and iron status. Nutr. J. 12 (1), 141–144. 10.1186/1475-2891-12-141 24188143 PMC3830559

[B66] McClung J. P. Scrimgeour A. G. (2005). Zinc: an essential trace element with potential benefits to soldiers. Mil. Med. 170 (12), 1048–1052. 10.7205/milmed.170.12.1048 16491946

[B67] MCO 10110.49 (2017). “Headquarters departments of the navy and the air force,” in Nutrition and menu standards for human performance optimization, 1–120.

[B68] Michlig S. Merlini J. M. Beaumont M. Ledda M. Tavenard A. Mukherjee R. (2016). Effects of TRP channel agonist ingestion on metabolism and autonomic nervous system in a randomized clinical trial of healthy subjects. Sci. Rep. 6 (5), 20795–20812. 10.1038/srep20795 26883089 PMC4756362

[B69] Mitchell H. H. Glickman N. Lambert E. H. Keeton R. W. Fahnestock M. K. (1946). The tolerance of man to cold as affected by dietary modification: carbohydrate versus fat and the effect of the frequency of meals. Am. J. Physiol. Content 146 (1), 84–96. 10.1152/ajplegacy.1946.146.1.84 21023298

[B70] Mora J. R. Iwata M. Von A. U. H. (2008). Vitamin effects on the immune system: vitamins A and D take centre stage. Nat. Rev. Immunol. 8 (9), 685–698. 10.1038/nri2378 19172691 PMC2906676

[B71] Muldoon M. F. Ryan C. M. Yao J. K. Conklin S. M. Manuck S. B. (2014). Long-chain omega-3 fatty acids and optimization of cognitive performance. Mil. Med. 179 (11), 95–105. 10.7205/MILMED-D-14-00168 25373092 PMC4734634

[B72] Naderi A. de Oliveira E. P. Ziegenfuss T. N. Willems M. E. T. (2016). Timing, optimal dose and intake duration of dietary supplements with evidence-based use in sports nutrition. J. Exerc Nutr. Biochem. 20 (4), 1–12. 10.20463/jenb.2016.0031 PMC5545206 28150472

[B73] Nielsen B. (1987). Does diet-induced thermogenesis change the preferred ambient temperature of humans? Eur. J. Appl. Physiol. Occup. Physiol. 56 (4), 474–478. 10.1007/BF00417778 3622491

[B74] Nieto Jimenez C. Cajigal Vargas J. Triantafilo Vladilo V. S. Naranjo Orellana J. (2018). Impact of hypothermic stress during special operations training of Chilean military Forces. Mil. Med. 183 (7–8), e193–e199. 10.1093/milmed/usx131 29425375

[B75] NIH (2015). A compilation of dietary supplement statements from the scientific report of the 2015 dietary guidelines advisory committee. Available at: https://ods.od.nih.gov/pubs/2015_DGAC_Scientific_Report_ODS_Compiled_DS_Statements.pdf.

[B76] O’Brien C. Mahoney C. Tharion W. J. Sils I. V. Castellani J. W. (2007). Dietary tyrosine benefits cognitive and psychomotor performance during body cooling. Physiol. Behav. 90 (2–3), 301–307. 10.1016/j.physbeh.2006.09.027 17078981

[B77] Palinkas L. A. Suedfeld P. (2008). Psychological effects of polar expeditions. Lancet. 371 (9607), 153–163. 10.1016/S0140-6736(07)61056-3 17655924

[B78] Pasiakos S. M. (2020). Nutritional requirements for sustaining health and performance during exposure to extreme environments. Annu. Rev. Nutr. 40, 221–245. 10.1146/annurev-nutr-011720-122637 32530730

[B79] Pecora F. Persico F. Argentiero A. Neglia C. Esposito S. (2020). The role of micronutrients in support of the immune response against viral infections. Nutrients 12 (10), 3198–3245. 10.3390/nu12103198 33092041 PMC7589163

[B80] Purkayastha S. S. Sharma R. P. Ilavazhagan G. Sridharan K. Ranganathan S. Selvamurthy W. (1999). Effect of vitamin C and E in modulating peripheral vascular response to local cold stimulus in man at high altitude. Jpn. J. Physiology 49, 159–167. 10.2170/jjphysiol.49.159 10393350

[B81] Radzikowska U. Rinaldi A. O. Sözener Z. Ç. Karaguzel D. Wojcik M. Cypryk K. (2019). The influence of dietary fatty acids on immune responses. Nutrients 11, 2990. 10.3390/nu11122990 31817726 PMC6950146

[B82] Rae C. Digney A. L. McEwan S. R. Bates T. C. (2003). Oral creatine monohydrate supplementation improves brain performance: a double-blind, placebo-controlled, cross-over trial. Proc. R. Soc. B Biol. Sci. 270 (1529), 2147–2150. 10.1098/rspb.2003.2492 PMC1691485 14561278

[B83] Rao S. S. C. Camilleri M. Hasler W. L. Maurer A. H. Parkman H. P. Saad R. (2011). Evaluation of gastrointestinal transit in clinical practice: position paper of the American and European Neurogastroenterology and Motility Societies. Neurogastroenterol. Motil. 23 (1), 8–23. 10.1111/j.1365-2982.2010.01612.x 21138500

[B84] Rawson E. S. Lieberman H. R. Walsh T. M. Zuber S. M. Harhart J. M. Matthews T. C. (2008). Creatine supplementation does not improve cognitive function in young adults. Physiol. Behav. 95 (1–2), 130–134. 10.1016/j.physbeh.2008.05.009 18579168

[B85] Ried K. Fakler P. (2014). Potential of garlic (Allium sativum) in lowering high blood pressure: mechanisms of action and clinical relevance. Integr. Blood Press Control 7, 71–82. 10.2147/IBPC.S51434 25525386 PMC4266250

[B86] Rosenzweig P. H. Volpe S. L. (1999). Iron, thermoregulation, and metabolic rate. Crit. Rev. Food Sci. Nutr. 39 (2), 131–148. 10.1080/10408399908500491 10198751

[B87] Ross A. C. Caballero B. H. Cousins R. J. Tucker K. L. Ziegler T. R. (2012). Modern nutrition in health and disease. Philadelphia, PA: Wolters Kluwer Health Adis ESP.

[B88] Saltykova M. M. (2019). Cold adaptation as a means of increasing antioxidant protection. Neurosci. Behav. Physiol. 49 (3), 323–330. 10.1007/s11055-019-00735-x

[B89] Sanders O. D. Rajagopal J. A. Rajagopal L. (2021). Menthol to induce non-shivering thermogenesis via TRPM8/PKA signaling for treatment of obesity. J. Obes. Metab. Syndr. 30 (1), 4–11. 10.7570/jomes20038 33071240 PMC8017329

[B90] Scholey A. B. French S. J. Morris P. J. Kennedy D. O. Milne A. L. Haskell C. F. (2010). Consumption of cocoa flavanols results in acute improvements in mood and cognitive performance during sustained mental effort. J. Psychopharmacol. 24 (10), 1505–1514. 10.1177/0269881109106923 19942640

[B91] Schwedhelm E. Maas R. Freese R. Jung D. Lukacs Z. Jambrecina A. (2008). Pharmacokinetic and pharmacodynamic properties of oral L‐citrulline and L‐arginine: impact on nitric oxide metabolism. Br. J. Clin. Pharmacol. 65 (1), 51–59. 10.1111/j.1365-2125.2007.02990.x 17662090 PMC2291275

[B92] Severo J. S. Morais J. B. S. De Freitas T. E. C. Andrade A. L. P. Feitosa M. M. Fontenelle L. C. (2019). The role of zinc in thyroid hormones metabolism. Int. J. Vitam. Nutr. Res. 89 (1–2), 80–88. 10.1024/0300-9831/a000262 30982439

[B93] Shepherd A. I. Costello J. T. Bailey S. J. Bishop N. Wadley A. J. Young-Min S. (2019). “Beet” the cold: beetroot juice supplementation improves peripheral blood flow, endothelial function, and anti-inflammatory status in individuals with Raynaud’s phenomenon. J. Appl. Physiol. 127 (5), 1478–1490. 10.1152/japplphysiol.00292.2019 31343948 PMC6879832

[B94] Shouk R. Abdou A. Shetty K. Sarkar D. Eid A. H. (2014). Mechanisms underlying the antihypertensive effects of garlic bioactives. Nutr. Res. 34 (2), 106–115. 10.1016/j.nutres.2013.12.005 24461311

[B95] Sullivan-Kwantes W. Haman F. Kingma B. R. M. Martini S. Gautier-Wong E. Chen K. Y. (2021). Human performance research for military operations in extreme cold environments. J. Sci. Med. Sport 24 (10), 954–962. 10.1016/j.jsams.2020.11.010 33358087

[B96] Sullivan-Kwantes W. Moes K. Limmer R. Goodman L. (2019). Finger cold-induced vasodilation test does not predict subsequent cold injuries: a lesson from the 2018 Canadian Forces Exercise. Temperature 6 (2), 142–149. 10.1080/23328940.2019.1574200 PMC6601413 31286025

[B97] Swann O. G. Kilpatrick M. Breslin M. Oddy W. H. (2020). Dietary fiber and its associations with depression and inflammation. Nutr. Rev. 78 (5), 394–411. 10.1093/nutrit/nuz072 31750916

[B98] Takano N. Kotani M. (1989). Influence of food intake on cold-induced vasodilatation of finger. Jpn. J. Physiol. 39 (5), 755–765. 10.2170/jjphysiol.39.755 2615036

[B99] Tanskanen M. M. Westerterp K. R. Uusitalo A. L. Atalay M. Häkkinen K. Kinnunen H. O. (2012). Effects of easy-to-use protein-rich energy bar on energy balance, physical activity and performance during 8 Days of sustained physical exertion. PLoS One 7 (10), e47771. 10.1371/journal.pone.0047771 23094083 PMC3475712

[B100] Taylor L. Watkins S. L. Marshall H. Dascombe B. J. Foster J. (2016). The impact of different environmental conditions on cognitive function: a focused review. Front. Physiol. 6 (JAN), 372–412. 10.3389/fphys.2015.00372 26779029 PMC4701920

[B101] Teichner W. H. (1958). Reaction time in the cold. J. Appl. Psychol. 42 (1), 54–59. 10.1037/h0049145

[B102] Teo L. Crawford C. Snow J. Deuster P. A. Bingham J. J. Gallon M. D. (2017a). Phytochemicals to optimize cognitive function for military mission-readiness: a systematic review and recommendations for the field. Nutr. Rev. 75, 49–72. 10.1093/nutrit/nux005 28969340

[B103] Teo L. Crawford C. Yehuda R. Jaghab D. Bingham J. J. Chittum H. K. (2017b). Omega-3 polyunsaturated fatty acids to optimize cognitive function for military mission-readiness: a systematic review and recommendations for the field. Nutr. Rev. 75, 36–48. 10.1093/nutrit/nux008 28969342

[B104] Thompson C. Wylie L. J. Fulford J. Kelly J. Black M. I. McDonagh S. T. J. (2015). Dietary nitrate improves sprint performance and cognitive function during prolonged intermittent exercise. Eur. J. Appl. Physiol. 115 (9), 1825–1834. 10.1007/s00421-015-3166-0 25846114

[B105] Tiollier E. Chennaoui M. Gomez-Merino D. Drogou C. Filaire E. Guezennec C. Y. (2007). Effect of a probiotics supplementation on respiratory infections and immune and hormonal parameters during intense military training. Mil. Med. 172 (9), 1006–1011. 10.7205/milmed.172.9.1006 17937368

[B106] Tremblay A. Arguin H. Panahi S. (2016). Capsaicinoids: a spicy solution to the management of obesity? Int. J. Obes. 40 (8), 1198–1204. 10.1038/ijo.2015.253 26686003

[B107] USDA (2019). FoodData Central Food Details: beverages, coffee, instant, regular, prepared with water. Available at: https://fdc.nal.usda.gov/food-details/174130/nutrients.

[B108] Valente A. Carrillo A. E. Tzatzarakis M. N. Vakonaki E. Tsatsakis A. M. Kenny G. P. (2015). The absorption and metabolism of a single L-menthol oral versus skin administration: effects on thermogenesis and metabolic rate. Food Chem. Toxicol. 86, 262–273. 10.1016/j.fct.2015.09.018 26429629

[B109] Vauzour D. (2012). Dietary polyphenols as modulators of brain functions: biological actions and molecular mechanisms underpinning their beneficial effects. Oxid. Med. Cell Longev. 2012, 914273. 10.1155/2012/914273 22701758 PMC3372091

[B110] Vieira S. F. Gonçalves V. M. F. Llaguno C. P. Macías F. Tiritan M. E. Reis R. L. (2022). On the bioactivity of Echinacea purpurea extracts to modulate the production of inflammatory mediators. Int. J. Mol. Sci. 23 (21), 13616–13625. 10.3390/ijms232113616 36362404 PMC9659013

[B111] Wakabayashi H. Sugiyama K. Suzuki S. Sakihama Y. Hashimoto M. Barwood M. J. (2023). Influence of acute beetroot juice supplementation on cold-induced vasodilation and fingertip rewarming. Eur. J. Appl. Physiol. 123 (3), 495–507. 10.1007/s00421-022-05071-6 36305974

[B112] Walsh N. P. Whitham M. (2006). Exercising in environmental extremes. Sport Med. 36 (11), 941–976. 10.2165/00007256-200636110-00003 17052132

[B113] Watanabe A. Kato N. Kato T. (2002). Effects of creatine on mental fatigue and cerebral hemoglobin oxygenation. Neurosci. Res. 42 (4), 279–285. 10.1016/s0168-0102(02)00007-x 11985880

[B114] Westerterp K. R. (2004). Diet induced thermogenesis. Nutr. Metab. 1, 5. 10.1186/1743-7075-1-5 PMC524030 15507147

[B115] Whiting S. Derbyshire E. Tiwari B. K. (2012). Capsaicinoids and capsinoids. A potential role for weight management? A systematic review of the evidence. Appetite 59 (2), 341–348. 10.1016/j.appet.2012.05.015 22634197

[B116] Whitney E. N. Rolfes S. R. (2013). Understanding nutrition. 13th ed. Wadsworth, OH: Cengage AU.

[B117] Wickham K. A. Steele S. W. Cheung S. S. (2021). Effects of acute dietary nitrate supplementation on cold-induced vasodilation in healthy males. Eur. J. Appl. Physiol. 121 (5), 1431–1439. 10.1007/s00421-021-04621-8 33620545

[B118] Wightman E. L. Haskell-Ramsay C. F. Thompson K. G. Blackwell J. R. Winyard P. G. Forster J. (2015). Dietary nitrate modulates cerebral blood flow parameters and cognitive performance in humans: a double-blind, placebo-controlled, crossover investigation. Physiol. Behav. 149, 149–158. 10.1016/j.physbeh.2015.05.035 26037632

[B119] Woelkart K. Linde K. Bauer R. (2008). Echinacea for preventing and treating the common cold. Planta Med. 74 (6), 633–637. 10.1055/s-2007-993766 18186015

[B120] Zhou E. Zhang L. He L. Xiao Y. Zhang K. Luo B. (2024). Cold exposure, gut microbiota and health implications: a narrative review. Sci. Total Environ. 916, 170060. 10.1016/j.scitotenv.2024.170060 38242473

